# Paraoxonase 1 gene variants concerning cardiovascular mortality in conventional cigarette smokers and non-smokers treated with hemodialysis

**DOI:** 10.1038/s41598-021-98923-8

**Published:** 2021-09-30

**Authors:** Alicja E. Grzegorzewska, Kamila Ostromecka, Monika K. Świderska, Paulina Adamska, Adrianna Mostowska, Paweł P. Jagodziński

**Affiliations:** 1 Department of Biochemistry and Molecular Biology, Poznan University of Medical Sciences, Święcickiego 6, 60-781 Poznań, Poland; 2 Department of Nephrology, Transplantology and Internal Diseases, Poznan University of Medical Sciences, Przybyszewskiego 49, 60-355 Poznań, Poland

**Keywords:** Genetics, Cardiology, Nephrology, Risk factors

## Abstract

Cigarette smoking effects might correspond with paraoxonase 1 gene (*PON1*) single nucleotide variants (SNVs). We investigated the association of *PON1* rs705379, rs854560, and rs662 with cardiovascular mortality in hemodialysis (HD) patients concerning conventional cigarette smoking. Cardiovascular, cardiac, coronary heart disease (CHD)- and non-CHD-related deaths were analyzed in 206 HD cigarette smokers and 659 HD non-smokers. P-values were adjusted for sex, age, and high-density lipoprotein cholesterol. Among all smokers, the rs705379 TT genotype was associated with cardiovascular (P = 0.028), cardiac (P = 0.046), and cardiac non-CHD-related (P = 0.001) mortality. Non-diabetic smokers showed similar qualitative significance to all smokers concerning mentioned death rates (P-values 0.011, 0.044, and 0.009, respectively). In diabetic non-smokers, the rs705379 T allele correlated with CHD-related deaths (P = 0.020). The rs854560 T allele was associated with lower cardiovascular mortality in non-diabetic smokers (P = 0.008). The rs854560 TT genotype showed a negative non-significant correlation with non-CHD-related cardiac death in all non-smokers (P = 0.079). In diabetic smokers, the rs662 G allele was associated with higher cardiac mortality (P = 0.005). In all non-smokers and non-diabetic non-smokers, the rs662 G correlated with cardiovascular deaths (P = 0.020 and P = 0.018, respectively). Genotyping *PON1* SNVs may help argue HD smokers harboring the rs705379 TT genotype or T allele and non-smokers possessing the rs662 G allele for prevention against cardiovascular diseases. These groups are more burdened genetically for cardiovascular mortality.

## Introduction

Paraoxonase 1 gene (*PON1*) single nucleotide variants (SNVs) as influencing paraoxonase 1 (PON-1) activity and concentration are associated with the peroxidation rate of high-density and low-density lipoproteins (HDL and LDL, respectively)^1–3^. The glutamine-containing PON-1 Q192 isoform (associated with the rs662 A allele of rs662 Q192R, 575A > G) was shown to protect LDL against oxidative modification more effectively than the arginine-containing PON-1 R192 isoform (associated with the rs662 G allele)^4,5^. Opposite results have also been demonstrated^6^. Two mentioned papers^4,6^ showed that the PON-1 Q192 isoform hydrolyses less paraoxon. *PON1* rs854560 (L55M) influences ribonucleic acid messenger (mRNA) levels. The *PON1* rs854560 T allele transcript related to methionine-containing PON-1 isoform is less stable than the A allele transcript coding leucine-containing PON-1 isoform. The rs854560 T allele bearers show lower PON-1 concentration and activity and are more susceptible to atherosclerosis and diseases associated with oxidative stress^7–9^. *PON1* rs705379 -108C > T expression is related to its impact on the formation of complexes between the specificity protein 1 (Sp1) transcription factor and the *PON1* promoter^10^. The *PON1* rs705379 CT and TT genotypes correspond with the lower PON-1 activity^11^.

*PON1* SNVs were directly related to atherosclerotic diseases, including coronary heart disease (CHD)^6,12,13^. Moreover, the rs662 G allele enhanced susceptibility to CHD (particularly myocardial infarction—MI) in end-stage non-insulin-dependent diabetes mellitus (NIDDM) patients^14^, cigarette smokers^15^, and older subjects^16^. However, other investigations did not show the relationship between the *PON1* SNVs and CHD^17^ or showing opposite results^18^.

Tobacco is responsible for 20% of CHD deaths^19^. Tobacco smoking is related to increased susceptibility to lipoprotein oxidation^20^. Cigarette smoke components, like acrolein, inhibit plasma PON-1 activity modifying its free thiols^21^ and decreasing HDL levels^22^. The cigarette packs smoked per year were associated with an increased MI risk in the rs662 AA homozygosity, related in this study to a low activity isoform of PON-1^15^. On the other hand, smokers with the rs662 GG genotype showed a higher atherogenic index and Framingham risk score than smoking and non-smoking AA + AG carriers. The rs662 GG genotype was discussed as associated with low arylesterase activity^23^. As one can see, the mentioned above data also do not provide thoroughly consistent results.

The HD patients have similar *PON1* SNVs distribution to a general population^24^, but their PON-1 activity is substantially lower^24,25^. A Japanese study showed that *PON1* rs662 and rs854560 exerted no impact on cardiovascular disease (CVD**)** mortality in 81 HD patients^26^.

From 14 to 25% of prevalent HD patients smoke cigarettes^27–29^. Tobacco smoking increases susceptibility to hemoglobin glycation^20,30^. It is important because approximately 50% of the incident HD receivers show diabetes mellitus (DM)^31^. The *PON1* rs705379 TT genotype was associated with NIDDM nephropathy, independently of demographic and clinical factors^29^.

This study planned to show differences between cigarette smokers and non-smokers among hemodialyzed patients who died from cardiovascular reasons. As cardiovascular effects of smoking might correspond to *PON1* SNVs, we investigated the association of *PON1* rs705379 (located in the *PON1* promoter region), rs854560, and rs662 (both located in the *PON1* coding region) with cardiovascular mortality in cigarette smokers and non-smokers. We assumed to document possible differences in the *PON1* impact on cardiovascular mortality concerning smoking status for obtaining additional arguments in anti-tobacco strategy in genetically burdened HD groups.

## Results

### Patient characteristics

Among HD patients who died from cardiovascular reasons, there were 82 smokers and 239 non-smokers. Concerning cardiovascular mortality, the 80% power could be obtained at 1.5 OR in dominant and additive models for rs662 and rs854560, and an additive model for rs705379 (Supplementary Table 1).

Smokers’ group comprised younger individuals at RRT onset and death, more men as a percent of the total, and more frequent atherogenic pattern of serum lipid profile defined as triglyceride (TG)/HDL cholesterol ratio ≥ 3.8. Both groups did not differ significantly in RRT duration, frequency of MI, body mass index, and the type of lipid-modifying medicines used (Table 1).

**Table 1:** Characteristics of HD patients who died from cardiovascular reasons concerning cigarette smoking status.

Tested parameters	Smokers (n = 82)	Non-smokers (n = 239)	P-value^b^
Male sex (n, %)	70 (85.3%)	91 (38.1%)	1.5e−13
Age at RRT onset (years)	57.5 (21.7–82.6)	67.6 (14.1–90.8)	7.293e−10
Age at death (years)	63.5 (31.1–86.3)	74.0 (26.8–95.9)	2.992e−10
RRT duration (years)	6.8 (0.7–28.0)	5.7 (0.3–28.7)	0.179
Renal transplantation in the course of RRT (n, %)	38 (46.3)	132 (55.2)	0.164
Lifespan with a functioning graft (years)	3.35 (0.15–20.9)	4.14 (0–19.0)	0.879
Number of cigarettes smoked per day	20 (2–60)	–	NA
NIDDM (n, %)	28 (34.1)	91 (38.1%)	0.525
Coronary heart disease (n, %)	48 (58.5%)	124 (52.1%)	0.297
Myocardial infarction (n, %)	29 (35.4%)	71 (29.8%)	0.340
Ischemic cerebral stroke (n, %)	12 (14.6%)	59 (24.7%)	0.058
Dry body mass (kg), n = 292	74.19 ± 15.22 n = 76	72.59 ± 14.79 n = 216	0.429^c^
Body mass index (kg/m^2^)^a^, n = 290	25.96 ± 4.51 n = 76	26.78 ± 4.51 n = 214	0.176^c^
TG/HDL cholesterol ratio ≥ 3.8 (n, %)	52 (63.4%)	113 (47.3%)	0.012
Total cholesterol (mg/dL)	171 (94–363)	170 (51–352)	0.996
HDL-cholesterol (mg/dL)	34.8 (17.3–103)	40 (7–103)	0.0004
LDL-cholesterol (mg/dL)	96.8 (32–255)	97 (13.3–350)	0.939
TG (mg/dL)	167.0 (48.8–652)	149.8 (40.0–856)	0.034
Non-HDL-cholesterol (mg/dL)	137 (57.9–282)	130 (32–313)	0.242
LDL/HDL cholesterol ratio	2.60 (0.71–7.10)	2.57 (0.50–9.46)	0.077
HDL/TC ratio	0.217 (0.102–0.540)	0.229 (0.061–0.586)	0.007
TG/HDL cholesterol ratio	4.9 (0.72–25.55)	3.79 (0.66–49.7)	0.003
**Lipid-modifying treatment (n, %)**	44 (53.7%)	106 (44.4%)	0.145
Statins (n, %)	38 (46.3%)	96 (40.2%)	0.238
Fibrates (n, %)	5 (6.1%)	4 (1.7%)	0.051^d^
Statin + ezetimibe (n, %)	1 (1.2%)	0 (0%)	0.256^d^

^a^Dry body mass was not calculated in 2 patients due to amputation of the leg(s); ^b^Mann–Whitney *U* test for quantitative variables and Pearson's Chi-squared test for qualitative variables; ^c^Student’s T-test; ^d^Fisher's Exact Test.

*HD* hemodialysis, *HDL* high-density lipoprotein, *LDL* low-density lipoprotein, *NA* not applicable, *NIDDM* non-insulin-dependent diabetes mellitus, *RRT* renal replacement therapy, *TG* triglycerides.

Conversion to SI units: to change cholesterol to mmol/L, divide by 38.6; triglycerides to mmol/L, by 88.5.

### Cardiovascular mortality

**Figure 1:**
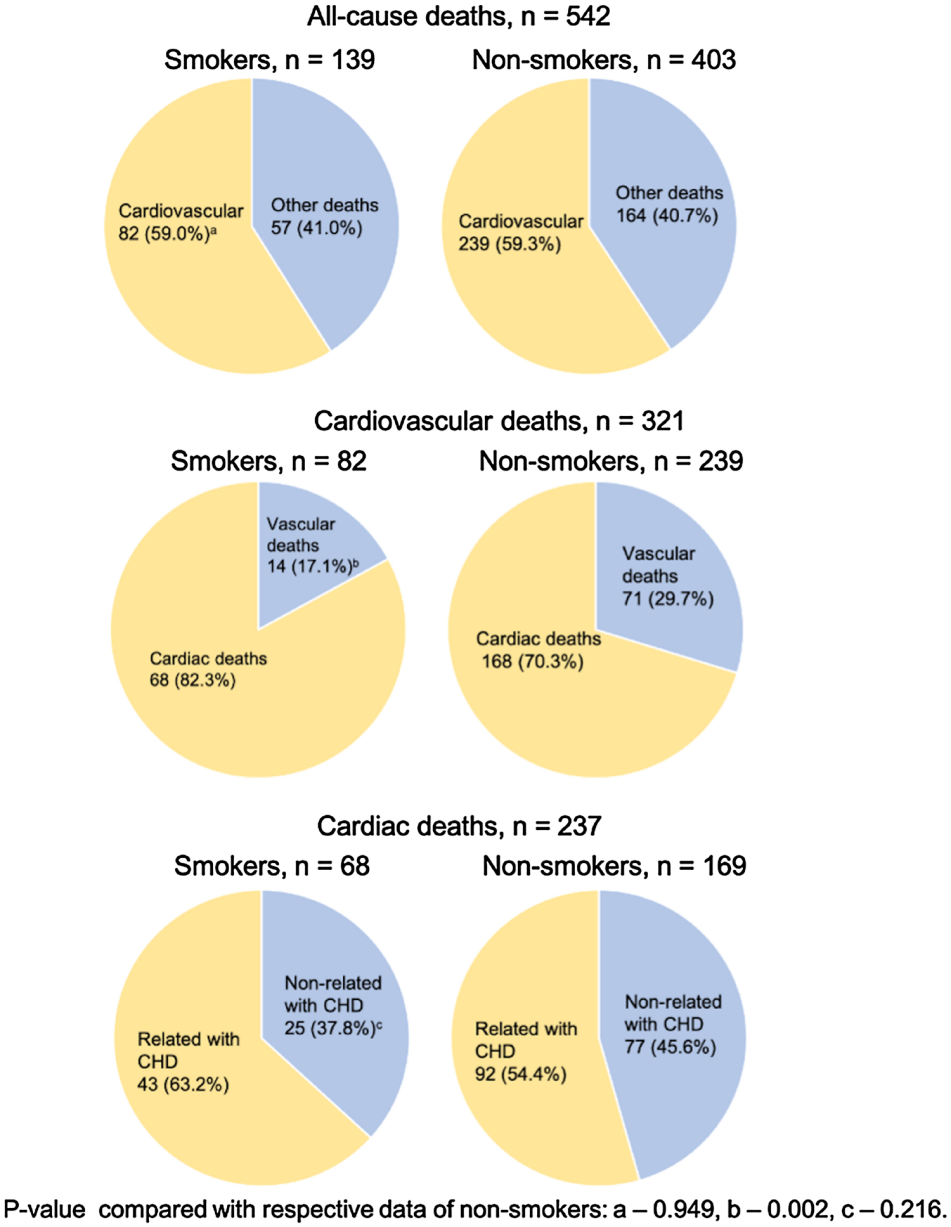
Prevalence of cardiovascular-related deaths in hemodialysis smokers and non-smokers.

All cardiac deaths were more frequent in smokers (Fig. 1), but the Kaplan–Meier cumulative proportion surviving was not different between smokers and non-smokers concerning cardiovascular mortality. NIDDM nephropathy patients presented worse cardiovascular and CHD-related cardiac survival than non-DM subjects (Table 2).

**Table 2:** Cardiovascular mortality in HD patients categorized by cigarette smoking status and occurrence of NIDDM nephropathy.

Cause of death	Log-rank test P-value	HR (95% CI)	Wald test on 1 df, P-value	Adjusted P-value^a^
**HD cigarette smokers and HD non-smokers as a reference group**				
Cardiovascular	0.4	0.899 (0.699–1.156)	0.69, 0.4	0.699
All cardiac	0.2	0.836 (0.629–1.110)	1.53, 0.2	0.437
Cardiac related with CHD	0.4	0.842 (0.585–1.212)	0.86, 0.4	0.490
Cardiac non-related with CHD	0.4	0.819 (0.516–1.301)	0.71, 0.4	0.730
**NIDDM nephropathy patients and non-diabetics as a reference group**				
Cardiovascular	0.01	1.348 (1.071–1.696)	6.47, 0.01	0.026
All cardiac	0.09	1.256 (0.961–1.640)	2.78, 0.1	0.143
Cardiac related with CHD	0.005	1.648 (1.154–2.354)	7.56, 0.006	0.016
Cardiac non-related with CHD	0.7	0.920 (0.602–1.405)	0.15, 0.7	0.765
**NIDDM nephropathy smokers and non-diabetic smokers as a reference group**				
Cardiovascular	0.3	1.306 (0.820–2.079)	1.26, 0.3	0.432
All cardiac	0.5	1.186 (0.706–1.992)	0.41, 0.5	0.753
Cardiac related with CHD	0.05	1.938 (0.986–3.809)	3.69, 0.05	0.235
Cardiac non-related with CHD	0.6	0.813 (0.347–1.909)	0.23, 0.6	0.853
**NIDDM nephropathy non-smokers and non-diabetic non-smokers as a reference group**				
Cardiovascular	0.02	1.360 (1.041–1.778)	5.08, 0.02	0.029
All cardiac	0.1	1.288 (0.940–1.765)	2.47, 0.1	0.116
Cardiac related with CHD	0.05	1.516 (0.989–2.323)	3.65, 0.06	0.050
Cardiac non-related with CHD	0.9	1.04 (0.638–1.695)	0.02, 0.9	0.898

^a^Adjustment for age, gender, and HDL-cholesterol.

Among HD cigarette smokers, the group showing NIDDM as a cause of end-stage renal disease (ESRD) did not differ in cardiovascular mortality from non-DM smokers (Supplementary Fig. 1). HD non-smokers demonstrated higher cardiovascular mortality if they had NIDDM nephropathy (Table 2). Cardiovascular mortality was not significantly different among NIDDM nephropathy patients or non-DM subjects categorized by smoking status (Supplementary Table 2).

### *PON1* SNVs

All selected patients (n = 865) underwent genotyping for *PON1* SNVs. Successful results were obtained in 818 patients for rs705379, 843—for rs854560, and 821—for rs662. The tested SNVs were distributed according to the Hardy–Weinberg equilibrium (HWE). P-values for HWE were 0.600 for *PON1* rs705379, 0.896 for *PON1* rs854560, and 0.902 for *PON1* rs662. *PON1* SNVs demonstrated weak linkage disequilibrium (LD, r^2^ < 0.3).

### *PON1* SNVs and patients’ survival probability

Due to a high number of survival-related analyses concerning *PON1* SNVs (Supplementary Tables 3–8), Table 3 shows only statistically significant (P < 0.05) results of these calculations. The Kaplan–Meier cumulative proportion surviving for analyses presented in Table 3 (for smokers and non-smokers) are shown in Supplementary Fig. 2A–L.

**Table 3:** Analysis of mortality concerning *PON1* polymorphisms in HD patients showing significant P-values by the log-rank test and Wald test.

Group of HD patients	Type of death	Deaths by inheritance modes	Log-rank test P-value	Wald test P-value	HR (Lower—Upper 95% CI), P-value	Adjusted^a^ HR P-value
All smokers, n = 193	Cardiovascular-related, n = 79	Recessive (*PON1* rs705379)	0.03	0.02	1.781 (1.066–2.975), 0.027	0.028
	All cardiac, n = 66	Recessive (*PON1* rs705379)	0.02	0.03	1.887 (1.076–3.306), 0.027	0.046
	Cardiac non-related with CHD, n = 25	Recessive (*PON1* rs705379)	6e-04	0.003	7.107 (1.962–25.75), 0.003	0.001
Smokers without NIDDM, n = 144	Cardiovascular-related, n = 52	Recessive (*PON1* rs705379)	0.008	0.01	2.390 (1.226–4.660), 0.011	0.011
	All cardiac, n = 45	Recessive (*PON1* rs705379)	0.02	0.02	2.414 (1.139–5.134), 0.022	0.044
	Cardiac non-related with CHD, n = 16	Recessive (*PON1* rs705379)	0.006	0.02	6.572 (1.417–30.47), 0.016	0.009
Smokers without NIDDM, n = 148	Cardiovascular-related, n = 54	Dominant (*PON1* rs854560)	0.04	0.04	0.446 (0.203–0.981), 0.045	0.008
Smokers with NIDDM, n = 49	All cardiac, n = 20	Dominant (*PON1* rs662)	0.03	0.04	3.410 (1.056–11.02), 0.040	0.005
All non-smokers, n = 624	Cardiovascular-related, n = 226	Dominant (*PON1* rs662)	0.02	0.02	1.372 (1.048–1.797), 0.022	0.020
Non-smokers without NIDDM, n = 470	Cardiovascular-related, n = 145	Dominant (*PON1* rs662)	0.01	0.01	1.531 (1.088–2.154), 0.015	0.018
All non-smokers, n = 645	Cardiac non-related with CHD, n = 75	Recessive (*PON1* rs854560)	0.04	0.04	0.435 (0.194–0.978), 0.044	0.079
Non-smokers with NIDDM, n = 156	Cardiac related with CHD, n = 40	Dominant (*PON1* rs705379)	0.02	0.02	2.511 (1.134–5.558), 0.023	0.020

*CI* coincidence interval, *HD* hemodialysis, *HR* hazard ratio, *NIDDM* non-insulin-dependent diabetes mellitus, *PON1* paraoxonase 1 gene.

^a^Adjusted for the male sex, age, and HDL-cholesterol.

Among all smokers, the rs705379 TT genotype was associated with all cardiovascular (P = 0.028, Supplementary Fig. 2A), all cardiac (P = 0.046, Supplementary Fig. 2B), and cardiac non-related with CHD (P = 0.001, Supplementary Fig. 2C) mortality. The rs705379 TT genotype smokers, who died from cardiac reasons, showed a higher MI frequency than CC + CT bearers (66.7% vs. 29.2%, P = 0.047). Concerning the rs705379 TT genotype, non-DM smokers showed similar qualitative significance like all smokers for all cardiovascular, all cardiac, and cardiac non-related with CHD death rates (P = 0.011, Supplementary Fig. 2D; P = 0.044, Supplementary Fig. 2E; and P = 0.009, Supplementary Fig. 2F; respectively). The rs854560 TT + AT genotypes were inversely associated with cardiovascular deaths in non-DM smokers (P = 0.008, Supplementary Fig. 2^2^). In DM smokers, the rs662 G allele was associated with a higher risk of cardiac mortality (P = 0.005, Supplementary Fig. 2H) (Table 3).

In all non-smokers and non-DM non-smokers, the rs662 G allele was associated with cardiovascular-related deaths (P = 0.020, Supplementary Fig. 2I and P = 0.018, Supplementary Fig. 2J, respectively). In DM non-smokers, the rs705379 T allele correlated with CHD's cardiac death (P = 0.020, Supplementary Fig. 2L) (Table 3).

## Discussion

Our study indicates that cigarette smoking is associated with more unfavorable characteristics of HD patients who died from cardiovascular diseases than HD non-smokers who also died from cardiovascular reasons. HD cigarette smokers started RRT at a younger age, died at a younger age, and presented more frequently atherogenic dyslipidemia due to lower serum HDL-cholesterol and higher serum TG levels. Cardiac deaths were more frequent in HD smokers. As expected, smokers were predominantly men. Differences in cardiovascular longevity were present inside smoking or non-smoking groups if patients` data were analyzed concerning *PON1* polymorphisms. Associations between *PON1* SNVs and cardiovascular mortality were more frequent and stronger in smokers than non-smokers.

The *PON1* rs705379 TT genotype was associated with higher cardiovascular and cardiac mortality, independently of age and NIDDM nephropathy^32^. The present study revealed that all HD smokers and non-DM smokers demonstrate increased cardiovascular, cardiac, and cardiac CHD non-related mortality if they possess the rs705379 TT genotype. In DM non-smokers, the rs705379 T allele correlated only with cardiac death related to CHD.

The *PON1* rs705379 TT genotype is associated with attenuated antioxidant, anti-inflammatory, anti-thrombosis, and anti-adhesion activities^1,33,34^. It may predispose to cardiac diseases^35,36^. The mean activity of the rs705379 T allele is 0.67–0.77-fold lower than that attributed to the C allele^37^. Under hyperglycemic conditions, circulating PON-1 activity is additionally diminished due to glycation of HDL-PON-1^38^. NIDDM patients have a decreased serum PON-1 activity^39^. The rs705379 TT genotype is associated with a 1.5-fold higher prevalence of end-stage NIDDM nephropathy than observed in the CT + CC genotype subjects, independently on smoking status^29^. All examined DM patients presented such type of nephropathy, and NIDDM nephropathy as a cause of ESRD was associated with higher cardiovascular mortality in subjects not categorized by smoking status. Therefore, in HD patients affected by end-stage NIDDM nephropathy, the rs705379 TT genotype could be at least partially related to cardiovascular deaths due to its association with this disease. PON-1 prevents or reduces cardiovascular complications in NIDDM patients, involving mechanisms such as decreasing plasma oxidized LDL concentrations, diminishing macrophage ability to uptake oxidized LDL and releasing reactive oxygen species, preventing macrophage proinflammatory responses, reducing foam cell generation, increasing macrophage cholesterol efflux, catabolizing homocysteine thiolactone, preventing oxidative inactivation of lecithin: cholesterol acyltransferase, inhibiting myeloperoxidase and monocyte chemotactic protein 1 activities, and preventing the glucose-induced glycoxidation^40^.

On the other hand, the rs705379 TT genotype relationship with cardiovascular mortality was revealed also in non-DM smokers, in whom it could not be related to NIDDM nephropathy. Non-DM current smokers are twice as likely as non-smokers to have increased glycated hemoglobin, but it remains in the pre-diabetes range^30^. Smoking itself enhances glycation to a lesser extent than DM does^30^. In HD non-DM smokers, decreased PON-1 activity in the TT genotype patients could be aggravated by glycation of HDL-PON-1, which is not present in HD non-smokers.

The current data showed that the rs662 G allele contributes to the higher susceptibility to cardiac deaths in HD smokers with NIDDM nephropathy, and cardiovascular mortality in all examined non-smokers and non-DM non-smokers. The rs662 GG genotype NIDDM nephropathy smokers and the rs662 G allele non-DM patients used a greater number of cigarettes or showed a higher frequency of smoking than those having the rs662 A allele or AA genotype^29^. Our results agree with meta-analyses indicating the association of the rs662 G allele with cardiovascular mortality in non-ESRD individuals^12,13^.

The *PON1* rs854560 T allele corresponded with a higher risk of CHD-related death in non-DM men^18^, an increased risk of CHD in Iranian patients with atherosclerosis^41^, the higher prevalence of ischemic cerebral stroke in the entire HD population^32^ and end-stage NIDDM nephropathy individuals^27^, but not with cardiovascular, cardiac, or vascular mortality in the whole HD subjects^32^. Surprisingly, in this study, the rs854560 T allele was inversely associated with cardiovascular mortality in non-DM smokers. Also, the TT genotype showed a negative correlation with cardiac death non-related to CHD in all non-smokers (no significant association after adjustment). The TT genotype was related in HD smokers to the lower number of cigarettes smoked per day^29^. This finding could be some explanation for better survival in the smoking HD group.

Results of genotyping *PON1* SNVs may help argue HD patients for prevention against cardiovascular diseases by rejecting or reducing cigarette smoking. According to the present findings, this conception is the most relevant in smokers harboring the rs705379 TT genotype or T allele. HD non-smokers possessing the rs662 G allele are also genetically burdened for cardiovascular mortality.

### Strengths and limitations of the study

*PON1* genetic variants determine serum PON-1 concentration and activity. Although serum PON-1 status influences oxidation, inflammation, and lipid properties, it is itself modified by several factors, which can be changing during a lifespan. Genetic variants are constant, so they may better predict mortality as a hard endpoint. Our study is the first one assessing *PON1* SNVs concerning cardiovascular deaths in HD patients, whose cigarette smoking status and DM coexistence were also considered significant contributors to mortality.

This study's limitation is its retrospective, although longitudinal, design instead of prospective observation, and a small number of patients in subgroups. The latter is challenging to obtain even a conventional statistical significance. Using multiplicity adjustment as Bonferroni corrected thresholds leads to a severe power loss in empirical analyzes of population-based association studies^42^. It has also occurred in this study. For Table 3, the significance level after Bonferroni correction is at α < 0.001 for the log-rank test (36 analyses performed) and α < 0.004 for the Wald test (12 analyzes computed). However, a situation in which Bonferroni analyses are recommended is when searching for significant associations without pre-established hypotheses^43^. Associations between *PON1* SNVs and cardiovascular death were documented already in CHD non-uremic patients^18^. In addition, our data show *PON1* SNV associations with cardiovascular mortality in several subgroups who died from cardiovascular reasons, which suggests that they are not accidental. Therefore, using conventional P-values of < 0.05 as indicating significance seems to be justified in our study, although we are conscious that P-values are not impressive. According to the Better Associations for Disease and GEnes (BADGE) system^44^, our genetic associations shown in Table 3 would have to confirm first-class or second-class BADGE analyses in independent population samples to be recognized as undoubtedly associated. Such high P-values are observed rarely in specific clinical groups. Thus, replication analyses on other HD groups or functional validation assays are warranted highly in future investigations.

## Materials and methods

### Patients

We have used deoxyribonucleic acid (DNA) samples for *PON1* genotyping from our DNA base. DNA probes were collected from January 2009 to June 2019 from Caucasian HD patients. At first, we selected subjects with known cigarette smoking status. HD patients were diagnosed as cigarette smokers if they smoked conventional cigarettes at least two years before RRT initiation and continued smoking through RRT. Non-smoking status was recognized if HD patients never smoked cigarettes or discontinued smoking at least five years before RRT started. Secondly, from DM patients identified in this primarily selected group, only NIDDM nephropathy subjects were enrolled. NIDDM nephropathy was diagnosed based on classical signs and symptoms. In the case of difficulties in NIDDM nephropathy's clinical diagnosis, a kidney biopsy confirmed or excluded this diagnosis. Among selected 865 HD subjects, there were 206 smokers and 659 non-smokers.

We analyzed demographic, clinical, and laboratory data collected during patients` lifespan and after that as needed and possible. Patients` outcome (survival on HD, death on HD, renal transplantation, a movement to not collaborating center) was checked in September 2020. In deceased individuals, death causes were registered based on medical documentation and categorized as cardiovascular, infection-related, cancer-related, and other or unknown. Among cardiovascular causes of death, we specified cardiac deaths related to CHD and those not associated with CHD.

### *PON1* genotyping

Three *PON1* polymorphisms were genotyped: rs662 (Q192R, 575A>G), rs854560 (L55M, 163A>T), and rs705379 (− 108C>T) using previously described methods^29,32^.

### Statistical analysis

Non-normally distributed variables by the Shapiro–Wilk test are presented as a median and range; data showing normal distribution are expressed as a mean ± standard deviation. Dichotomous variables are shown as a percentage of the total number. Mann–Whitney *U* test or Student's T-test were used to compare quantitative variables. Pearson's Chi-squared test or Fisher's exact test was applied to compare qualitative variables, as appropriate.

The power of the study was calculated using the Genetic Association Study (GAS) Power Calculator (http://csg.sph.umich.edu/abecasis/gas_power_calculator/index.html) with the following inputs: number of cases (HD smokers who died from cardiovascular reasons) = 100, number of controls = 240 (HD non-smokers who died from cardiovascular diseases), significance level = 0.05, prevalence = 55%^45^.

HWE was calculated by the Chi-squared test (df = 1, P > 0.05 for agreement). LD between *PON1* SNVs was computed using the Haploview 4.2 software (http://www.broad.mit.edu/mpg/haploview/).

Survival analysis covered a period from the RRT start to death on regular HD treatment. CVD-related deaths were analyzed by the *PON1* genotypes and in modes of inheritance using the Kaplan–Meier method with the log-rank test. Inheritance modes (dominant, recessive) were created concerning variant alleles as the risk alleles^46^. We have made three assumptions regarding the log-rank test, namely that the censoring is unrelated to the outcome, the survival probabilities are the same for participants recruited early and late in the study, and the events were occurred at the times specified. If the analyzes yielded the log-rank test P-values < 0.05, the Cox regression was applied. For the Cox regression model, we have tested two assumptions: the proportional hazards and the linear relationship between the log hazard and each covariate using graphical methods. We considered the significance in the Cox analyses if the Wald test P-value was < 0.05 (Supplementary Tables 3–8). Results, significant in the Cox regression, were adjusted for sex, age, and HDL-cholesterol. The latter was the serum lipid parameter, which yielded the most significant difference between smokers and non-smokers (Table 1). Hazard ratio (HR) with 95% confidence interval (CI) and P-values were computed. If the adjusted P-values were < 0.05, we considered a significant association between tested *PON1* SNV and the defined cause of cardiovascular death.

All analyses we performed using Graph-Pad InStat 3.10, 32 bit for Windows, created July 9, 2009 (GraphPad Software, Inc., San Diego, California, United States), Statistica version 13, 2017 (TIBCO Software Inc., 3307 Hillview Avenue Palo Alto, CA 94,304 USA) and R 3.6.1 (R Foundation for Statistical Computing, Vienna, Austria)^47^.

### Ethical issues

The Institutional Review Board of the Poznan University of Medical Sciences, Poland, approved our study's design. All methods we carried out by relevant guidelines and regulations (Declaration of Helsinki).

### Consent to participate

The informed written consent was obtained at the blood collection for DNA extraction from all study participants or their parents if participants were under 18 years.

### Conference presentation

Abstract of this paper has been accepted as a Mini-Oral at the 58th ERA-EDTA Congress, organized from June 5 to 8, 2021, in collaboration with the German Society of Nephrology (Deutsche Gesellschaft für Nephrologie).

## Supplementary Information

Supplementary Information.

## Data Availability

All data are available for any reasonable request from the first author.
