# Supplementary material for: Paraoxonase 1 gene variants concerning cardiovascular mortality in conventional cigarette smokers and non-smokers treated with hemodialysis
Source: Sci Rep. 2021 Sep 30;11:19467. doi: 10.1038/s41598-021-98923-8 (PMC8484566; doi:10.1038/s41598-021-98923-8)

Paraoxonase 1 Gene Variants Concerning Cardiovascular Mortality in Conventional Cigarette Smokers and Non-smokers Treated with Hemodialysis

Alicja E. Grzegorzewska, Professor^1^*, Kamila Ostromecka, student^1^, Monika K. Świderska, PhD^2^, Paulina Adamska, student^1^, Adrianna Mostowska, Professor^1^, Paweł P. Jagodziński, Professor^1^

^1^ Department of Biochemistry and Molecular Biology, Poznan University of Medical Sciences, 60-781 Poznań, Święcickiego 6, Poland. E-mails: [alicja_grzegorzewska@yahoo.com](mailto:alicja_grzegorzewska@yahoo.com), k[amila.ostromecka@onet.pl](mailto:amila.ostromecka@onet.pl), [p.adamska01@gmail.com](mailto:p.adamska01@gmail.com), [amostowska@wp.pl](mailto:amostowska@wp.pl), pjagodzi@ump.edu.pl

^2^ Department of Nephrology, Transplantology and Internal Diseases, Poznan University of Medical Sciences, 60-355 Poznań, Przybyszewskiego 49, Poland. E-mail: monika.swi@gmail.com

^*^Corresponding author: Prof. Alicja E. Grzegorzewska. Department of Biochemistry and Molecular Biology, Poznan University of Medical Sciences, 60-781 Poznań, Święcickiego 6, Poland. Tel. mobile (48) 696 08 44 87; Fax: (48) 618546510; E-mail: alicja_grzegorzewska@yahoo.com

Supplementary material

Supplementary Table 1. The expected power for 1.00 – 1.75 ORs in associations analyses between cardiovascular mortality in smokers and non-smokers in hemodialysis patients

|  |  |  |  |  |  |  |  |  |  |
| --- | --- | --- | --- | --- | --- | --- | --- | --- | --- |
|  |  |  | **The expected power** | | |  |  |  |  |
|  | **Allele** | **Genotype** |  | **Genetic model** | |  |  |  |  |
| **rs number** | **frequency^a^** | **relative risk** | **Additive** | **Dominant** | **Recessive** |  |  |  |  |
| **rs662** | 0.2815 | 1.00 | 0.050 | 0.050 | 0.050 |  |  |  |  |
|  |  | 1.25 | 0.730 | 0.470 | 0.129 |  |  |  |  |
|  |  | 1.50 | 0.996 | 0.924 | 0.358 |  |  |  |  |
|  |  | 1.75 | 1.000 | 0.996 | 0.649 |  |  |  |  |
| **rs854560** | 0.3660 | 1.00 | 0.050 | 0.050 | 0.050 |  |  |  |  |
|  |  | 1.25 | 0.763 | 0.416 | 0.202 |  |  |  |  |
|  |  | 1.50 | 0.997 | 0.871 | 0.584 |  |  |  |  |
|  |  | 1.75 | 1.000 | 0.986 | 0.885 |  |  |  |  |
| **rs705379** | 0.4803 | 1.00 | 0.050 | 0.050 | 0.050 |  |  |  |  |
|  |  | 1.25 | 0.762 | 0.360 | 0.323 |  |  |  |  |
|  |  | 1.50 | 0.995 | 0.713 | 0.815 |  |  |  |  |
|  |  | 1.75 | 1.000 | 0.917 | 0.984 |  |  |  |  |
|  |  |  |  |  |  |  |  |  |  |
| ^a^ The Genome Aggregation Database v.3.1.1 [gnomAD, genome sequencing data, population: European (non-Finnish)]. | | | | | | | | | |
|  | | | | | | | | | |
| Supplementary Table 2. Cardiovascular mortality in end-stage NIDDM nephropathy patients or non-diabetic patients categorized inside each group by cigarette smoking status   \| Cause of death \| Log-rank test P-value \| HR (95% CI) \| Wald test on 1 df, P-value \| Adjusted P-value^a^ \| \| --- \| --- \| --- \| --- \| --- \| \| NIDDM nephropathy patients, non-smokers as a reference group \| \| \| \| \| \| Cardiovascular \| 0.6 \| 0.878 (0.570 – 1.352) \| 0.35, 0.6 \| 0.712 \| \| All cardiac \| 0.5 \| 0.830 (0.507 – 1.361) \| 0.54, 0.5 \| 0.604 \| \| Cardiac related with CHD \| 0.8 \| 1.092 (0.583 – 2.049) \| 0.08, 0.8 \| 0.900 \| \| Cardiac non-related with CHD \| 0.3 \| 0.682 (0.305 – 1.526) \| 0.87, 0.4 \| 0.520 \| \| Non-diabetic patients, non-smokers as a reference group \| \| \| \| \| \| Cardiovascular \| 0.7 \| 0.940 (0.687 – 1.286) \| 0.15, 0.7 \| 0.824 \| \| All cardiac \| 0.5 \| 0.889 (0.626 – 1.261) \| 0.44, 0.5 \| 0.900 \| \| Cardiac related with CHD \| 0.5 \| 0.842 (0.534 – 1.327) \| 0.55, 0.5 \| 0.633 \| \| Cardiac non-related with CHD \| 1.0 \| 1.004 (0.566 – 1.784) \| 0, 1.0 \| 0.465 \|   a – adjustment for age, gender, and HDL-cholesterol | | | | | | | | |  |

Supplementary Table 3. Analysis of HD smokers` mortality by log rank test and Wald test concerning *PON1* rs854560 variants

| Group of HD patients | Type of death | Deaths by *PON1* rs854560 genotypes or inheritance mode | Log rank test P-value | Cox regression (Wald test P-value) |
| --- | --- | --- | --- | --- |
| All smokers, n = 198 | All cardiac, n = 67 | AA, n = 31 ; AT, n = 33; TT, n = 3  P-value for HWE 0.113 | 0.3 |  |
|  |  | Dominant | 0.1 |  |
|  |  | Recessive | 0.7 |  |
|  | Cardiac related with CHD, n = 42 | AA, n = 19; AT, n = 20; TT, n = 3  P-value for HWE 0.460 | 0.4 |  |
|  |  | Dominant | 0.2 |  |
|  |  | Recessive | 0.7 |  |
|  | Cardiac non-related with CHD, n = 25 | AA, n = 12; AT, n =13 ; TT, n = 0  P-value for HWE 0.079 | 0.3 |  |
|  |  | Dominant | 0.3 |  |
|  |  | Recessive | NA |  |
|  | Cardiovascular-related, n = 81 | AA, n = 34; AT, n = 41; TT, n = 6  P-value for HWE 0.178 | 0.3 |  |
|  |  | Dominant | 0.1 |  |
|  |  | Recessive | 0.7 |  |
| Smokers with NIDDM, n = 50 | All cardiac, n = 21 | AA, n = 5; AT, n = 14; TT, n = 2  P-value for HWE 0.098 | 0.8 |  |
|  |  | Dominant | 0.7 |  |
|  |  | Recessive | 0.5 |  |
|  | Cardiac related with CHD, n = 12 | AA, n = 1; AT, n = 9; TT, n = 2  P-value for HWE 0.077 | 0.003 | 0.6 |
|  |  | Dominant | 9E-04 | 1.0 |
|  |  | Recessive | 0.3 |  |
|  | Cardiac non-related with CHD, n = 9 | AA, n = 4; AT, n = 5; TT, n = 0  P-value for HWE 0.249 | 0.4 |  |
|  |  | Dominant | 0.4 |  |
|  |  | Recessive | NA |  |
|  | Cardiovascular-related, n = 27 | AA, n = 6; AT, n = 18; TT, n = 3  P-value for HWE 0.069 | 0.6 |  |
|  |  | Dominant | 0.7 |  |
|  |  | Recessive | 0.4 |  |
| Smokers without NIDDM, n = 148 | All cardiac, n = 46 | AA, n = 26; AT, n = 19; TT, n = 1  P-value for HWE 0.242 | 0.1 |  |
|  |  | Dominant | 0.05 |  |
|  |  | Recessive | 0.7 |  |
|  | Cardiac related with CHD, n = 30 | AA, n = 18; AT, n = 11; TT, n = 1  P-value for HWE 0.660 | 0.1 |  |
|  |  | Dominant | 0.04 | 0.05 |
|  |  | Recessive | 0.7 |  |
|  | Cardiac non-related with CHD, n = 16 | AA, n = 8; AT, n = 8; TT, n = 0  P-value for HWE 0.182 | 0.4 |  |
|  |  | Dominant | 0.4 |  |
|  |  | Recessive | NA |  |
|  | Cardiovascular-related, n = 54 | AA, n = 28; AT, n = 23; TT, n = 3  P-value for HWE 0.536 | 0.1 |  |
|  |  | Dominant | 0.04 | 0.04 |
|  |  | Recessive | 1.0 |  |

Abbreviations: CHD - coronary heart disease, df – degrees of freedom, HD – hemodialysis, NIDDM - non-insulin-dependent diabetes mellitus, *PON1* - paraoxonase 1 gene

Supplementary Table 4. Analysis of HD smokers` mortality by log rank test and Wald test concerning *PON1* rs705379 variants

| Group of HD patient | Type of death | Deaths by *PON1* rs705379 genotypes or inheritance mode | Log rank test P-value | Cox regression (Wald test P-value) |
| --- | --- | --- | --- | --- |
| All smokers, n = 193 | All cardiac, n = 66 | CC, n =20 ; CT, n = 28; TT, n = 18  P-value for HWE 0.221 | 0.07 |  |
|  |  | Dominant | 0.2 |  |
|  |  | Recessive | 0.02 | 0.03 |
|  | Cardiac related with CHD, n = 41 | CC, n = 8; CT, n = 10; TT, n = 13  P-value for HWE 0.060 | 0.6 |  |
|  |  | Dominant | 0.6 |  |
|  |  | Recessive | 0.3 |  |
|  | Cardiac non-related with CHD, n = 25 | CC, n = 12 ; CT, n = 8; TT, n = 5  P-value for HWE 0.127 | 0.003 | 0.01 |
|  |  | Dominant | 0.4 |  |
|  |  | Recessive | 6e-04 | 0.003 |
|  | Cardiovascular-related, n = 79 | CC, n = 24 ; CT, n = 34; TT, n = 21  P-value for HWE 0.220 | 0.07 |  |
|  |  | Dominant | 0.2 |  |
|  |  | Recessive | 0.03 | 0.02 |
| Smokers with NIDDM, n = 49 | All cardiac, n = 21 | CC, n = 5 ; CT, n = 8; TT, n = 8  P-value for HWE 0.309 | 0.7 |  |
|  |  | Dominant | 0.5 |  |
|  |  | Recessive | 0.6 |  |
|  | Cardiac related with CHD, n = 12 | CC, n = 1 ; CT, n = 4; TT, n = 7  P-value for HWE 0.700 | 0.7 |  |
|  |  | Dominant | 0.5 |  |
|  |  | Recessive | 0.5 |  |
|  | Cardiac non-related with CHD, n = 9 | CC, n = 4 ; CT, n = 4; TT, n = 1  P-value for HWE 1.000 | 0.2 |  |
|  |  | Dominant | 0.7 |  |
|  |  | Recessive | 0.09 |  |
|  | Cardiovascular-related, n = 27 | CC, n = 6 ; CT, n = 13; TT, n = 8  P-value for HWE 0.869 | 0.8 |  |
|  |  | Dominant | 0.5 |  |
|  |  | Recessive | 0.8 |  |
| Smokers without NIDDM, n = 144 | All cardiac, n = 45 | CC, n = 15 ; CT, n = 20; TT, n = 10  P-value for HWE 0.502 | 0.06 |  |
|  |  | Dominant | 0.6 |  |
|  |  | Recessive | 0.02 | 0.02 |
|  | Cardiac-related with CHD, n = 29 | CC, n = 7 ; CT, n = 16; TT, n = 6  P-value for HWE 0.573 | 0.5 |  |
|  |  | Dominant | 0.7 |  |
|  |  | Recessive | 0.3 |  |
|  | Cardiac non-related with CHD, n = 16 | CC, n = 8 ; CT, n = 4; TT, n = 4  P-value for HWE 0.062 | 0.02 | 0.05 |
|  |  | Dominant | 0.5 |  |
|  |  | Recessive | 0.006 | 0.02 |
|  | Cardiovascular-related, n = 52 | CC, n = 18 ; CT, n = 21; TT, n = 13  P-value for HWE 0.183 | 0.03 | 0.04 |
|  |  | Dominant | 0.6 |  |
|  |  | Recessive | 0.008 | 0.01 |

Abbreviations: CHD - coronary heart disease, df – degrees of freedom, HD – hemodialysis, NIDDM - non-insulin-dependent diabetes mellitus, *PON1* - paraoxonase 1 gene

Supplementary Table 5. Analysis of HD smokers` mortality by log rank test and Wald test concerning *PON1* rs662 variants

| Group of HD patient | Type of death | Deaths by *PON1* rs662 genotypes or inheritance mode | Log rank test P-value | Cox regression (Wald test P-value) |
| --- | --- | --- | --- | --- |
| All smokers, n = 197 | All cardiac, n = 64 | AA, n = 28; AG, n = 31; GG, n = 5  P-value for HWE 0.368 | 0.4 |  |
|  |  | Dominant | 0.3 |  |
|  |  | Recessive | 0.3 |  |
|  | Cardiac related with CHD, n = 40 | AA, n = 19 ; AG, n = 18; GG, n = 3  P-value for HWE 0.651 | 0.3 |  |
|  |  | Dominant | 1.0 |  |
|  |  | Recessive | 0.1 |  |
|  | Cardiac non-related with CHD, n = 24 | AA, n = 9 ; AG, n = 13; GG, n = 2  P-value for HWE 0.367 | 0.2 |  |
|  |  | Dominant | 0.1 |  |
|  |  | Recessive | 0.9 |  |
|  | Cardiovascular-related, n = 78 | AA, n = 39 ; AG, n = 34; GG, n = 5  P-value for HWE 0.500 | 0.6 |  |
|  |  | Dominant | 0.7 |  |
|  |  | Recessive | 0.3 |  |
| Smokers with NIDDM, n = 49 | All cardiac, n = 20 | AA, n = 13 ; AG, n = 7; GG, n = 0  P-value for HWE 0.343 | NA |  |
|  |  | Dominant | 0.03 | 0.04 (1 df) |
|  |  | Recessive | NA |  |
|  | Cardiac related with CHD, n = 12 | AA, n = 9 ; AG, n = 3; GG, n = 0  P-value for HWE 0.621 | NA |  |
|  |  | Dominant | 0.4 |  |
|  |  | Recessive | NA |  |
|  | Cardiac non-related with CHD, n = 8 | AA, n = 4 ; AG, n = 4; GG, n = 0  P-value for HWE 0.346 | NA |  |
|  |  | Dominant | 0.007 | 1.0 (1 df) |
|  |  | Recessive | NA |  |
|  | Cardiovascular-related, n = 26 | AA, n = 18 ; AG, n = 8; GG, n = 0  P-value for HWE 0.354 | NA |  |
|  |  | Dominant | 0.2 |  |
|  |  | Recessive | NA |  |
| Smokers without NIDDM, n = 148 | All cardiac, n = 44 | AA, n = 15 ; AG, n = 24; GG, n = 5  P-value for HWE 0.319 | 0.4 |  |
|  |  | Dominant | 0.8 |  |
|  |  | Recessive | 0.2 |  |
|  | Cardiac related with CHD, n = 28 | AA, n = 10 ; AG, n = 15; GG, n = 3  P-value for HWE 0.450 | 0.2 |  |
|  |  | Dominant | 0.8 |  |
|  |  | Recessive | 0.06 |  |
|  | Cardiac non-related with CHD, n = 16 | AA, n = 5 ; AG, n = 9; GG, n = 2  P-value for HWE 0.507 | 0.9 |  |
|  |  | Dominant | 0.7 |  |
|  |  | Recessive | 1.0 |  |
|  | Cardiovascular-related, n = 52 | AA, n = 21 ; AG, n = 26; GG, n = 5  P-value for HWE 0.451 | 0.4 |  |
|  |  | Dominant | 0.9 |  |
|  |  | Recessive | 0.2 |  |

Abbreviations: CHD - coronary heart disease, df – degrees of freedom, HD – hemodialysis, NIDDM - non-insulin-dependent diabetes mellitus, *PON1* - paraoxonase 1 gene

Supplementary Table 6. Analysis of HD non-smokers` mortality by log rank test and Wald test concerning *PON1* rs854560 variants

| Group of HD patients | Type of death | Deaths by *PON1* rs854560 genotypes or inheritance mode | Log rank test P-value | Cox regression (Wald test P-value) |
| --- | --- | --- | --- | --- |
| All non-smokers, n = 645 | All cardiac, n = 167 | AA, n = 74 ; AT, n = 70; TT, n = 23  P-value for HWE 0.330 | 0.6 |  |
|  |  | Dominant | 0.9 |  |
|  |  | Recessive | 0.4 |  |
|  | Cardiac related with CHD, n = 92 | AA, n = 36; AT, n = 43; TT, n = 13  P-value for HWE 0.978 | 0.7 |  |
|  |  | Dominant | 0.5 |  |
|  |  | Recessive | 0.5 |  |
|  | Cardiac non-related with CHD, n = 75 | AA, n = 38; AT, n =27; TT, n = 10  P-value for HWE 0.157 | 0.1 |  |
|  |  | Dominant | 0.3 |  |
|  |  | Recessive | 0.04 | 0.04 (1 df) |
|  | Cardiovascular-related, n = 235 | AA, n = 100; AT, n =96; TT, n = 39  P-value for HWE 0.057 | 0.7 |  |
|  |  | Dominant | 0.9 |  |
|  |  | Recessive | 0.4 |  |
| Non-smokers with NIDDM, n = 163 | All cardiac, n = 65 | AA, n = 27; AT, n = 25; TT, n = 13  P-value for HWE 0.119 | 0.4 |  |
|  |  | Dominant | 0.6 |  |
|  |  | Recessive | 0.5 |  |
|  | Cardiac related with CHD, n = 42 | AA, n = 15; AT, n = 18; TT, n = 9  P-value for HWE 0.418 | 0.2 |  |
|  |  | Dominant | 0.09 |  |
|  |  | Recessive | 0.7 |  |
|  | Cardiac non-related with CHD, n = 23 | AA, n = 12; AT, n = 7; TT, n = 4  P-value for HWE 0.140 | 0.2 |  |
|  |  | Dominant | 0.1 |  |
|  |  | Recessive | 0.1 |  |
|  | Cardiovascular-related, n = 88 | AA, n = 35; AT, n = 35; TT, n = 18  P-value for HWE 0.103 | 0.8 |  |
|  |  | Dominant | 1.0 |  |
|  |  | Recessive | 0.5 |  |
| Non-smokers without NIDDM, n = 482 | All cardiac, n = 102 | AA, n = 47; AT, n = 45; TT, n = 10  P-value for HWE 0.871 | 0.7 |  |
|  |  | Dominant | 0.7 |  |
|  |  | Recessive | 0.4 |  |
|  | Cardiac related with CHD, n = 50 | AA, n = 21; AT, n = 25; TT, n = 4  P-value for HWE 0.355 | 0.9 |  |
|  |  | Dominant | 0.8 |  |
|  |  | Recessive | 0.8 |  |
|  | Cardiac non-related with CHD, n = 52 | AA, n = 26; AT, n = 20; TT, n = 6  P-value for HWE 0.483 | 0.3 |  |
|  |  | Dominant | 0.8 |  |
|  |  | Recessive | 0.2 |  |
|  | Cardiovascular-related, n = 147 | AA, n = 65; AT, n = 61; TT, n = 21  P-value for HWE 0.284 | 0.6 |  |
|  |  | Dominant | 0.7 |  |
|  |  | Recessive | 0.3 |  |

Abbreviations: CHD - coronary heart disease, df – degrees of freedom, HD – hemodialysis, NIDDM - non-insulin-dependent diabetes mellitus, *PON1* - paraoxonase 1 gene

Supplementary Table 7. Analysis of HD non-smokers` mortality by log rank test and Wald test concerning *PON1* rs705379 variants

| Group of HD patient | Type of death | Deaths by *PON1* rs705379 genotypes or inheritance mode | Log rank test P-value | Cox regression (Wald test P-value) |
| --- | --- | --- | --- | --- |
| All non-smokers, n = 625 | All cardiac, n = 165 | CC, n =47; CT, n = 76; TT, n = 42  P-value for HWE 0.317 | 0.3 |  |
|  |  | Dominant | 0.9 |  |
|  |  | Recessive | 0.1 |  |
|  | Cardiac related with CHD, n = 89 | CC, n = 25; CT, n = 39; TT, n = 25  P-value for HWE 0.244 | 0.2 |  |
|  |  | Dominant | 0.1 |  |
|  |  | Recessive | 0.09 |  |
|  | Cardiac non-related with CHD, n = 76 | CC, n = 22 ; CT, n = 37; TT, n = 17  P-value for HWE 0.847 | 0.1 |  |
|  |  | Dominant | 0.06 |  |
|  |  | Recessive | 0.8 |  |
|  | Cardiovascular-related, n = 230 | CC, n = 66 ; CT, n = 107; TT, n = 57  P-value for HWE 0.301 | 0.6 |  |
|  |  | Dominant | 1.0 |  |
|  |  | Recessive | 0.3 |  |
| Non-smokers with NIDDM, n = 156 | All cardiac, n = 64 | CC, n = 19; CT, n = 29; TT, n = 16  P-value for HWE 0.463 | 0.4 |  |
|  |  | Dominant | 0.3 |  |
|  |  | Recessive | 0.3 |  |
|  | Cardiac related with CHD, n = 40 | CC, n = 11; CT, n = 17; TT, n = 12  P-value for HWE 0.344 | 0.06 |  |
|  |  | Dominant | 0.02 | 0.02 (1 df) |
|  |  | Recessive | 0.4 |  |
|  | Cardiac non-related with CHD, n = 24 | CC, n = 8 ; CT, n = 12; TT, n = 4  P-value for HWE 0.889 | 0.3 |  |
|  |  | Dominant | 0.2 |  |
|  |  | Recessive | 0.8 |  |
|  | Cardiovascular-related, n = 86 | CC, n = 23; CT, n = 39; TT, n = 24  P-value for HWE 0.389 | 0.8 |  |
|  |  | Dominant | 0.6 |  |
|  |  | Recessive | 0.5 |  |
| Non-smokers without NIDDM, n = 469 | All cardiac, n = 101 | CC, n = 28; CT, n = 47; TT, n = 26  P-value for HWE 0.488 | 0.3 |  |
|  |  | Dominant | 0.7 |  |
|  |  | Recessive | 0.2 |  |
|  | Cardiac related with CHD, n = 49 | CC, n = 14; CT, n = 22; TT, n = 13  P-value for HWE 0.477 | 0.4 |  |
|  |  | Dominant | 0.6 |  |
|  |  | Recessive | 0.2 |  |
|  | Cardiac-non related with CHD, n = 52 | CC, n = 14; CT, n = 25; TT, n = 13  P-value for HWE 0.783 | 0.5 |  |
|  |  | Dominant | 0.3 |  |
|  |  | Recessive | 0.8 |  |
|  | Cardiovascular-related, n = 144 | CC, n = 43; CT, n = 68; TT, n = 33  P-value for HWE 0.541 | 0.5 |  |
|  |  | Dominant | 0.8 |  |
|  |  | Recessive | 0.3 |  |

Abbreviations: CHD - coronary heart disease, df – degrees of freedom, HD – hemodialysis, NIDDM - non-insulin-dependent diabetes mellitus, *PON1* - paraoxonase 1 gene

Supplementary Table 8. Analysis of HD non-smokers` mortality by log rank test and Wald test concerning *PON1* rs662 variants

| Group of HD patient | Type of death | Deaths by *PON1* rs662 genotypes or inheritance mode | Log rank test P-value | Cox regression (Wald test P-value) |
| --- | --- | --- | --- | --- |
| All non-smokers, n = 624 | All cardiac, n = 159 | AA, n = 86; AG, n = 66; GG, n = 7  P-value for HWE 0.197 | 0.2 |  |
|  |  | Dominant | 0.1 |  |
|  |  | Recessive | 0.3 |  |
|  | Cardiac related with CHD, n = 87 | AA, n = 49; AG, n = 34; GG, n = 4  P-value for HWE 0.531 | 0.7 |  |
|  |  | Dominant | 0.5 |  |
|  |  | Recessive | 0.5 |  |
|  | Cardiac non-related with CHD, n = 72 | AA, n = 37; AG, n = 32; GG, n = 3  P-value for HWE 0.222 | 0.3 |  |
|  |  | Dominant | 0.1 |  |
|  |  | Recessive | 0.3 |  |
|  | Cardiovascular-related, n = 226 | AA, n = 129; AG, n = 89; GG, n = 8  P-value for HWE 0.118 | 0.04 | 0.04 (2 df) |
|  |  | Dominant | 0.02 | 0.02 (1 df) |
|  |  | Recessive | 0.1 |  |
| Non-smokers with NIDDM, n = 154 | All cardiac, n = 59 | AA, n = 34; AG, n = 24; GG, n = 1  P-value for HWE 0.158 | 0.7 |  |
|  |  | Dominant | 0.9 |  |
|  |  | Recessive | 0.4 |  |
|  | Cardiac related with CHD, n = 37 | AA, n = 22; AG, n = 15; GG, n = 0  P-value for HWE 0.122 | NA |  |
|  |  | Dominant | 0.6 |  |
|  |  | Recessive | NA |  |
|  | Cardiac non-related with CHD, n = 22 | AA, n = 12; AG, n = 9; GG, n = 1  P-value for HWE 0.670 | 0.5 |  |
|  |  | Dominant | 0.7 |  |
|  |  | Recessive | 0.3 |  |
|  | Cardiovascular-related, n = 81 | AA, n = 45; AG, n = 34; GG, n = 2  P-value for HWE 0.128 | 0.3 |  |
|  |  | Dominant | 0.8 |  |
|  |  | Recessive | 0.1 |  |
| Non-smokers without NIDDM, n = 470 | All cardiac, n = 100 | AA, n = 52; AG, n = 42; GG, n = 6  P-value for HWE 0.513 | 0.2 |  |
|  |  | Dominant | 0.07 |  |
|  |  | Recessive | 0.3 |  |
|  | Cardiac related with CHD, n = 50 | AA, n = 27; AG, n = 19; GG, n = 4  P-value for HWE 0.799 | 0.6 |  |
|  |  | Dominant | 0.5 |  |
|  |  | Recessive | 0.3 |  |
|  | Cardiac non-related with CHD, n = 50 | AA, n = 25; AG, n = 23; GG, n = 2  P-value for HWE 0.238 | 0.2 |  |
|  |  | Dominant | 0.06 |  |
|  |  | Recessive | 0.6 |  |
|  | Cardiovascular-related, n = 145 | AA, n = 84; AG, n = 55; GG, n = 6  P-value for HWE 0.416 | 0.04 | 0.04 (2 df) |
|  |  | Dominant | 0.01 | 0.01 (1 df) |
|  |  | Recessive | 0.3 |  |

Abbreviations: CHD - coronary heart disease, df – degrees of freedom, HD – hemodialysis, NIDDM - non-insulin-dependent diabetes mellitus, *PON1* - paraoxonase 1 gene

Supplementary Fig. 1. Prevalence of cardiovascular deaths in hemodialysis smokers and non-smokers categorized by NIDDM nephropathy (Yes vs. No)


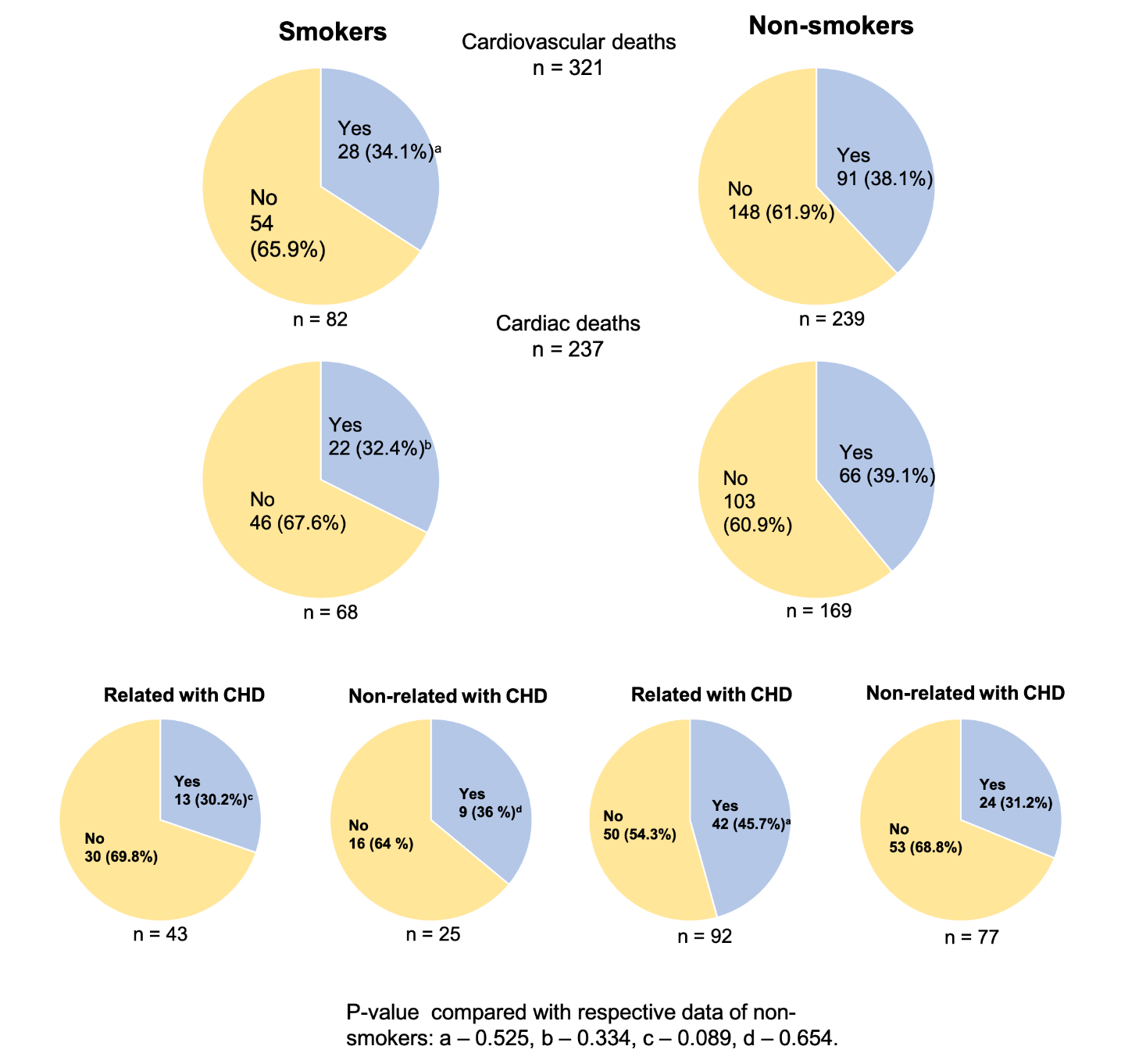


Supplementary Fig. 2. The Kaplan-Meier cumulative proportion surviving for analyses shown in Tables 3 and 4 in the main text


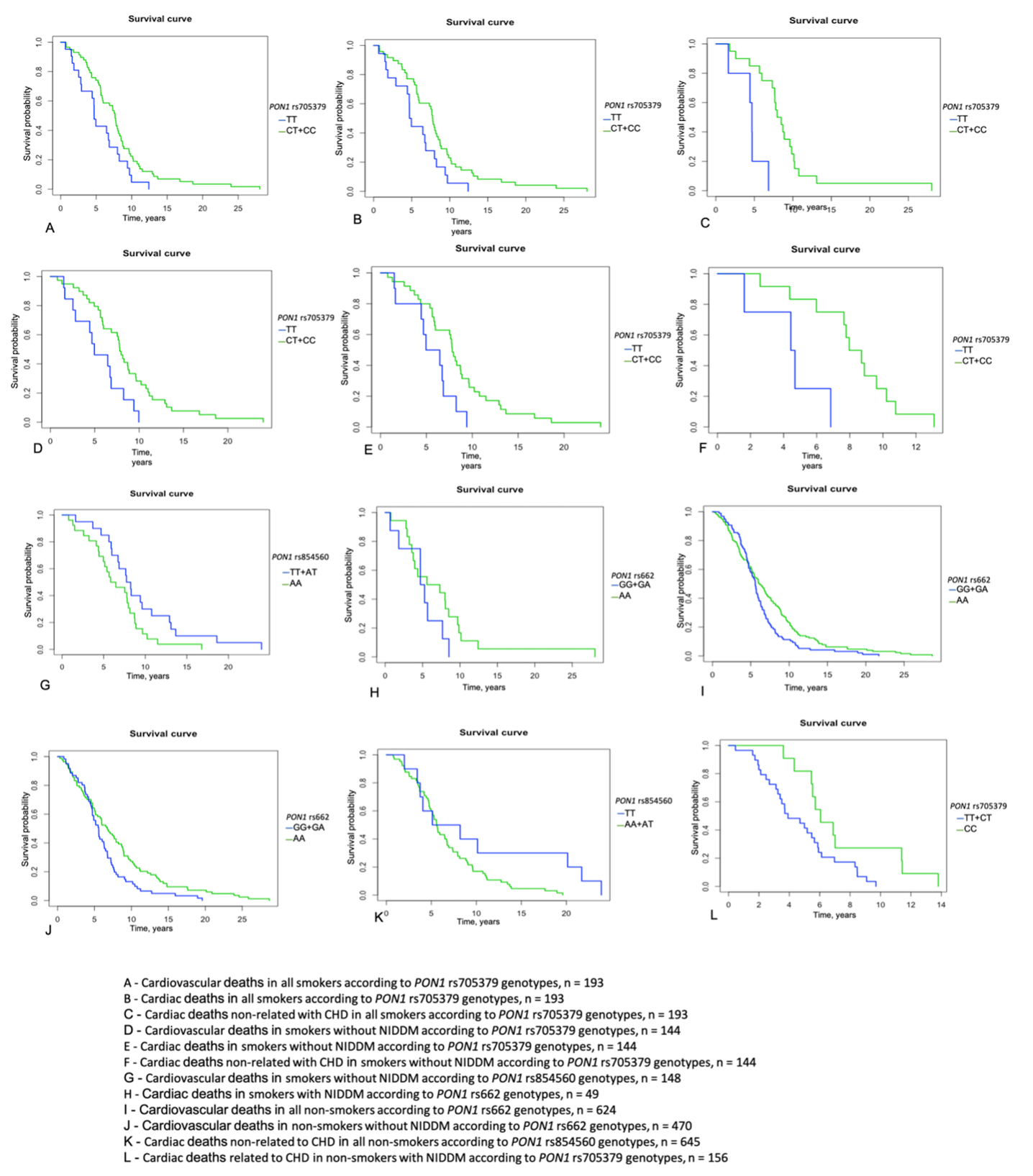

Supplement: Supplementary file 1 — Supplementary Information. [file 41598_2021_98923_MOESM1_ESM.docx]
